# Childhood maltreatment associated with elevated Herpes simplex virus 1 antibody concentrations in severe mental illness

**DOI:** 10.1192/j.eurpsy.2025.707

**Published:** 2025-08-26

**Authors:** D. Andreou, N. E. Steen, K. N. Jørgensen, T. Ueland, I. Drabløs, L. A. Wortinger, O. A. Andreassen, R. H. Yolken, I. Agartz

**Affiliations:** 1 Division of Mental Health and Substance Abuse, Diakonhjemmet Hospital; 2 Institute of Clinical Medicine, University of Oslo; 3 Division of Mental Health and Addiction, Oslo University Hospital; 4 Research Institute of Internal Medicine, Oslo University Hospital, Rikshospitalet, Oslo, Norway; 5 Stanley Division of Developmental Neurovirology, Department of Pediatrics, Johns Hopkins University School of Medicine, Baltimore, MD, United States

## Abstract

**Introduction:**

Adverse childhood events have been associated with immune aberrations. Herpes simplex virus 1 (HSV1), a neurotropic pathogen, establishes persistent infection after primary exposure. Elevated HSV1 immunoglobulin (IgG) levels have been found in HSV1-infected patients with severe mental illness (SMI), which likely reflects immune dysregulation.

**Objectives:**

We assessed childhood maltreatment and HSV1 IgG concentrations in adult patients with SMI and healthy controls. We hypothesized that maltreatment would be associated with elevated HSV1 IgG concentrations reflecting a failure in immune competence, and that such a putative association would be stronger in or even restricted to patients.

**Methods:**

We included 448 adult patients with SMI (mean age=31 years, 48% women, 46% HSV1 seropositive), i.e., 259 patients with schizophrenia spectrum and 189 patients with bipolar disorders, and 271 adult healthy controls (mean age=32, 41% women, 46% HSV1 seropositive). We assessed childhood maltreatment with the Childhood Trauma Questionnaire (CTQ), a 28-item retrospective self-report scale. We evaluated circulatory HSV1 IgG concentrations, expressed as continuous and dichotomous measures. In our main analyses, we applied sex- and age-adjusted multiple regressions on HSV1 IgG concentrations.

**Results:**

In patients with SMI (p=0.002) but not in healthy controls (p=0.203), CTQ total score was associated with HSV1 IgG seropositivity. Among seropositive patients (p<0.001) but not healthy controls (p=0.957), CTQ total score was associated with increased HSV1 IgG concentrations. Post-hoc analysis among seropositive patients showed that the five subscale scores for physical (p=0.002), sexual (p=0.019) and emotional abuse (p=0.002), and physical (p=0.012) and emotional neglect (p=0.016) were all associated with increased HSV1 IgG concentrations.

**Conclusions:**

Among patients with SMI, childhood maltreatment is associated with an increased risk of HSV1 infection. Further, among HSV1-infected patients, maltreatment is associated with elevated HSV1 antibody concentrations which may reflect a link between childhood adverse experiences and an immune system dysregulation.

**Disclosure of Interest:**

D. Andreou: None Declared, N. E. Steen: None Declared, K. N. Jørgensen: None Declared, T. Ueland: None Declared, I. Drabløs: None Declared, L. Wortinger: None Declared, O. Andreassen Consultant of: HealthLytix, Speakers bureau of: Speaker’s honoraria from Lundbeck and Sunovion, R. Yolken: None Declared, I. Agartz Speakers bureau of: Speaker’s honoraria from Lundbeck

